# Hypoxia inducible factors in hepatocellular carcinoma

**DOI:** 10.18632/oncotarget.17358

**Published:** 2017-04-21

**Authors:** Chu Chen, Tao Lou

**Affiliations:** ^1^ Department of Internal Medicine, Fourth Affiliated Hospital of Zhejiang University, School of Medicine, Yiwu, 322000, Zhejiang, China

**Keywords:** liver cancer, hepatoma, HIF, hypoxia, therapy

## Abstract

Hepatocellular carcinoma is one of the most prevalent and lethal cancers with limited therapeutic options. Pathogenesis of this disease involves tumor hypoxia and the activation of hypoxia inducible factors. In this review, we describe the current understanding of hypoxia signaling pathway and summarize the expression, function and target genes of hypoxia inducible factors in hepatocellular carcinoma. We also highlight the recent progress in hypoxia-targeted therapeutic strategies in hepatocellular carcinoma and discuss further the future efforts for the study of hypoxia and/or hypoxia inducible factors in this deadly disease.

## INTRODUCTION

Hepatocellular carcinoma (HCC) is the most common form of liver cancer (70%–90%). As the 2nd leading cause of global cancer mortality, HCC endangers over 780,000 new patients per year [1]. The etiological factors of HCC consist of, but not limited to, viral infections (hepatitis B and C viruses), alcohol intake, smoking, and many host factors such like cirrhosis, hemochromatosis, non-alcoholic steatohepatitis as well as diabetes [1–4]. Current options for HCC treatment include hepatectomy, transarterial chemoembolization, thermal or chemical ablation, liver transplantation, radiation and chemotherapy [4–6]. However, HCC behaves highly refractory to most anti-cancer therapies. Five year survival rate of HCC patients remains dismal [7].

HCC appears frequently as multiple nodules which are resulted from either intrahepatic metastasis or independent multicentric development [8]. Albeit both normal liver and HCC are highly vascularized, rapid growth of tumor cells within these nodules scavenges a substantial amount of oxygen, therefore often producing a hypoxic microenvironment. Indeed, HCC is one of the most hypoxic tumors with median oxygen level as low as 0.8% [9]. Inadequate intratumoral oxygen level is known to trigger a vast array of molecular and cellular responses which will influence tumor aggressiveness and therapeutic response. Hypoxia inducible factors (HIFs) are critical to sense intratumoral oxygen tension and mediate subsequently the activation of hypoxia response, thus representing as potential anti-cancer targets [10].

In this review, we are going to discuss the functional relevance of hypoxia and HIFs in HCC, and summarize recent progresses in therapeutic targeting of hypoxia pathway in this deadly disease.

### Hypoxia signaling pathway

**Figure 1:**
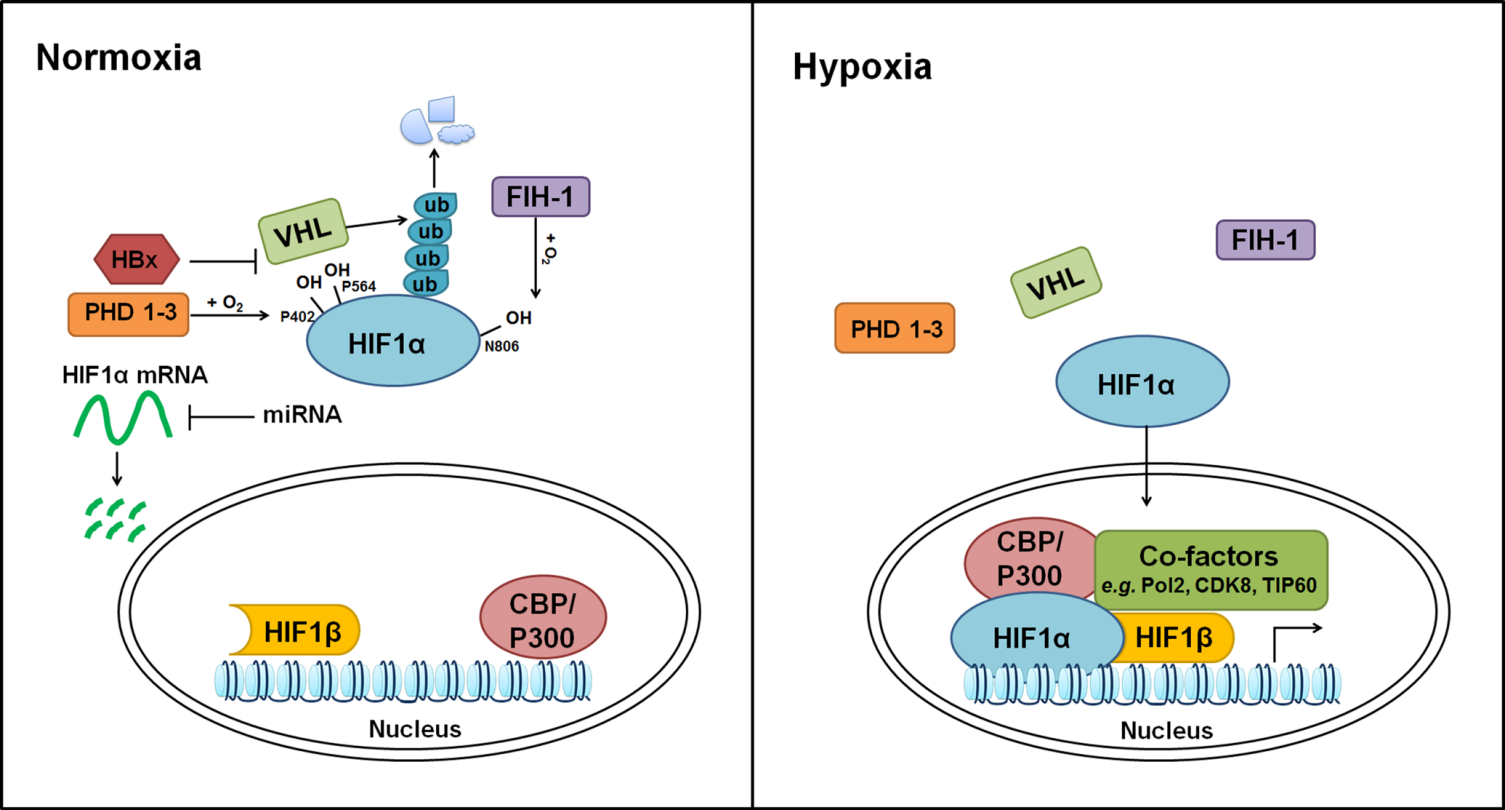
Regulation of hypoxia pathway HIF1α and HIF1β are used as examples. At post-transcriptional level, HIF1α mRNA is repressed by miR-199a-5p, miR-338-3p, miR-93 and miR-122. Under normoxia, HIF1α protein is hydroxylated at P402 and P564 by PHDs and subsequently degraded by pVHL through the Ubiquitin–Proteasome Pathway. Additionally, asparaginyl hydroxylation of HIF1α by FIH-1 at N806 impairs its interaction with CBP/P300. Hypoxia blocks the hydroxylation and proteasomal degradation of HIF1α, leading to its stabilization and nuclear translocation. Within nucleus, HIF1α forms heterodimer with HIF1β, and activate the expression of hypoxia responsive genes with the help from additional transcriptional co-factors, such as CBP/P300, Pol2, CDK8, and TIP60.

HIF system is implemented in hypoxia-responsive pathway. This system is composed of α-subunits (HIFα, including HIF1α, HIF2α/EPAS1 and HIF3α) and β-subunits (HIFβ, including HIF1β/ARNT1, ARNT2 and ARNT3). Among these proteins, the function and activity of HIF1α, HIF2α and HIF1β are relatively well-studied. Under normoxia (normal oxygen supply), HIFα is constitutively degraded and maintained at very low basal activities. Prolyl hydroxylation of HIFα (e.g. Pro 402 and 564 in human HIF1α) by prolyl hydroxylase domain-containing proteins (PHD1, PHD2 and PHD3) potentiates its subsequent recognition, ubiquitination and proteasomal degradation by an E3 ligase, von Hippel-Lindau tumor suppressor protein (pVHL). Moreover, asparaginyl hydroxylation of HIFα (e.g. Asn 803 in human HIF1α) by factor inhibiting HIF (FIH) blocks its interaction with transcriptional co-activators, CREB-binding protein (CBP) and p300 [11, 12]. Under hypoxia, hydroxylation and proteasomal degradation of HIFα are impaired due to lack of sufficient oxygen. Stabilized HIFα is then translocates into nucleus, hetero-dimerizes with HIFβ and binds core hypoxia-response element (HRE, 5′-(A/G)CGTG-3′) [13] (Figure 1). HIF1α binds preferentially to permissive chromatins where are positive for histone acetylation, H3K4me3, BRD4 and RNA-Pol2 signals [14, 15]. HIF1α utilizes CDK8-Mediator for its interaction with super-elongation-complex, thus activating the paused RNA-Pol2 and elevating the expression of HIF target genes [14]. Recently, Perez-Perri JI, et al. identified TIP60 complex as an additional co-activator to facilitate HIF1α-dependent chromatin modification and RNA-Pol2 activation [16]. Moreover, the activation of HIF1α transcriptional potency is associated with its interaction with CH1 domains of CBP and p300 [17, 18]. Loss of these CH1 domains in mouse embryonic fibroblasts showed deficiency of CBP or p300 loading on HIF targeted genes and affected the expression of 35%–50% HIF responsive genes [19]. Interestingly, while PHD proteins-dependent hydroxylation primes HIFα for pVHL-mediated destruction, HIF1α induces pVHL, PHD2, and PHD3, suggesting a feedback regulatory mechanism [20–26].

HIF activation has been associated with transcriptional induction of its target genes in most studies. However, using mouse hepatoma cells, Johnson AB. *et al*. reported that hypoxia may induce a general transcriptional repression via altering histone modifications [27]. To date, although transcriptome analysis of hypoxia response in HCC tissues/cells is limited, accumulating evidences support that hypoxia regulates cancer epigenetics. In HepG2, a human HCC cell line, hypoxia increased overall methylation of H3K4, H3K9 and H3K27. Meanwhile, several jumonji-domain histone demethylases, such as JMJD1A, JMJD2B and JMJD2C, were identified as HIF1α targets and were highly induced under hypoxia. The upregulation of histone demethylases may serve as an adaptive strategy to compensate the hypoxic stress and maintain the methylation homeostasis in HCC cells [28]. In parallel to histone modification, hypoxia also downregulated the overall level of 5-hydroxymethylcytosine (5-hmC) in HepG2 and Hep3B cells [29]. Since the Tet methylcytosine dioxygenase (TET) proteins convert 5-methylcytosine (5-mC) to 5-hmC, it would be interesting to study the connections among hypoxia, TET proteins and 5-hmC in HCC.

### Expression of HIFs in normal liver and HCC

In murine liver and hepatoma cells, *Hif1a* transcript containing exon 1.2 but not exon 1.1 was selectively expressed [30]. Diethyl nitrosamine (DEN) and palmitic acid elevate the transcription of HIF1α in primary hepatocytes [31]. In murine liver, nuclear HIF1α can be detected in normal hepatocytes under normoxic condition, and protein levels of HIF1α, HIF2α and HIF1β are hypoxia-inducible [32, 33]. The induction of HIF1α protein was also seen in the liver of Hepatitis B virus X (HBx) transgenic mice [34].

In human HCC samples, the protein level of HIF1α is significantly elevated and associated with worse prognosis [35–37]. Moreover, HIF1α expression in primary HCC tumors is an independent prognosis factor for overall survival of patients after receiving abdominal metastatic lymph node external beam radiotherapy [38]. However, the expression of HIF1α mRNA, HIF2α protein and HIF3α protein shows some variation [37, 39, 40] (Table 1).

**Table 1:** Expression of HIFs in HCC and their association with clinical outcomes

Gene	Expression	Prognostic potential	Reference
HIF1α	Predominantly expressed in tumor tissues (72/126) while less stained in peritumoral tissues (7/126)	Positively associated with worse disease-free survival and overall survival	[35]
	Highly expressed at both mRNA (42/110) and protein (39/110) levels	Positively associated with worse disease-free survival and overall survival after surgery	[37]
	Positively associated with HBx protein in HCC samples	Positively associated with worse disease-free survival and overall survival after surgery	[41]
	Positive in HCC samples (32/60)	Positively associated with shorter disease-free survival	[42]
	Positive in HCC samples (212/406)	Positively associated with higher probability of disease recurrence and worse overall survival after surgery.	[43]
	Highly expressed in 30/69 HCC samples (11/30 samples show nuclear staining)	Positively associated with the responses of abdominal metastatic lymph nodes to external beam radiotherapy, local recurrence and cancer-specific deaths	[38]
HIF2α	Expressed in peritumoral regions (60/126) while less expressed in tumor tissues (17/126)	No correlation	[35]
	Positive in HCC and adjacent noncancerous tissues Negative in normal liver tissues	Positively associated with shorter overall survival	[36]
	Lower in HCC on average	Negatively associated with worse survival	[44]
HIF3α	Inconsistently expressed between HCC and adjacent tissues	No correlation	[45]

The expression of HIFs is governed by both transcriptional and post-transcriptional mechanisms. In HCC cells, NF-κB subunits p50 and p65, but not c-Rel, bound the HIF1α promoter and elevated HIF1α transcription [46]. In addition to well-established oxygen-sensitive regulation of HIFs, HBx stabilized HIF1α through inhibiting its binding with pVHL [47]. According to recent studies, HBx protein also bridged the interaction between HIF1α and MTA1/HDAC complex, leading to deacetylation of HIF1α. The deacetylation of HIF1α interfered with its binding to PHDs and VHL, and subsequently stabilized/activated HIF1α protein in HBV-associated HCC cells [34, 41]. Furthermore, microRNA network appears as a new regulatory layer of HIF turnover (see review [48]). For example, miR-199a-5p, miR-338-3p, miR-93 and miR-122 have been shown to repress the expression of HIF1α in HCC cells [46, 49–51] (Figure 1).

### Functional relevance of HIFs in HCC

Cell line models of both human HCC and murine hepatoma origins have been applied directly to evaluate the function of HIFs in HCC. In human HepG2 and SK-Hep-1 HCC cells, HIF1α silencing strongly inhibited their anchorage independent growth, but did not affect their basal proliferation [52]. siRNA-mediated knockdown of HIF2α in HepG2 cells impaired cell cycle progression in the presence of CoCl_2_ and reduced cell proliferation both *in vitro* and *in vivo* [53]. In spheroid culture condition, dual-silencing of HIF1α and HIF2α diminished the growth of HepG2 cells, whereas knockdown of either HIF1α or HIF2α increased spheroid size and decreased caspase-3 activity [54]. Rat hepatoma cells with deficiency in HIF1β subunit showed much reduced tumorigenic ability in athymic mice, when compared with their wildtype counterparts [55].

Genetic data from mouse models also support the functional importance of of HIFs in HCC (Table 2). By using liver-specific transposon-based insertional mutagenesis method, *Hif1a* genomic region was identified with recurrent insertions in murine HCC models [56], indicating the potential involvement of aberrant HIF1α expression in HCC development. Knockout of *Hif1a* sensitized hepatoma cells to etoposide treatment in a transgenic murine model with hepatocyte-specific expression of SV40 large T oncogene, but did not affect the initiation and progression of murine HCCs [52]. Interestingly, liver-specific HIF1α overexpressing potentiated the development of HCC-promoting M2 macrophages [31]. Besides, myeloid HIF2α appeared to be required for liver tumor progression. Mice lacking HIF2α in myeloid cells showed decreased infiltration of tumor-associated macrophages in HCC and delayed tumor progression [57].

**Table 2:** Functional study of HIFs in mouse HCC models

Approach	Genetic background	Cells	Results	Reference
Transposon-based insertional mutagenesis	Mixed	Hepatocyte	Recurrent insertions in *Hif1a* genomic region	[56]
*Hif1α* knockout	C57Bl/6J	Hepatoma cells with SV40 large T antigen expression	Sensitized the cells to etoposide treatment	[52]
HIF1α overexpression	C57BL/6	Hepatocyte	Increased percentage of M2 macrophages	[31]
HIF2α deficiency	Mixed	Myeloid cell	Decreased infiltration of TAM in diethylnitrosamine (DEN) induced hepatocellular carcinoma	[57]

HIF1α has been shown to promote HCV replication in hepatocytes and to potentiate the migration of hepatoma cells [58]. HIF-induced VEGF expression promoted HCV entry by causing depolarization and reducing tight junction in hepatocytes [59, 60].

### HIF target genes in HCC and their functional contributions

**Figure 2:**
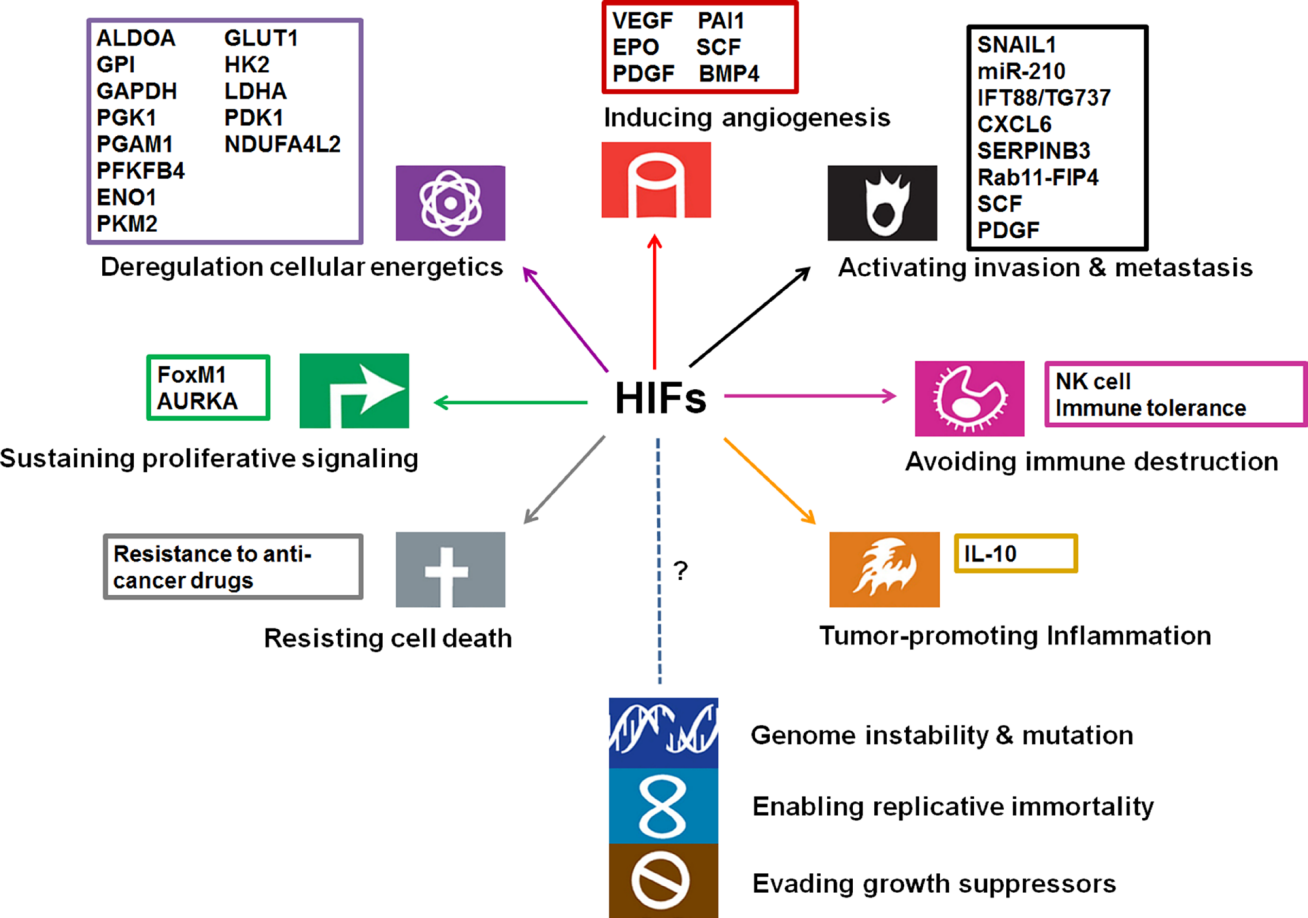
Involvement of HIFs and their targets in cancer hallmarks (modified from the original figure from Hanahan and Weinberg [113]) The function of HIFs has been implicated in promoting angiogenesis, invasion/metastasis, proliferation, glycolysis, therapeutic resistance, inflammation, and immune evasion.

To date, albeit the direct genetic evidences supporting the involvement of HIFs await further characterization, a growing body of literatures has reported the identification and function of HIF target genes in HCC. The functional relevance of hypoxia/HIF target genes have been implicated in most cancer hallmarks (Figure 2).

### Angiogenesis

HCC is highly vascularized as a result of up-regulation of angiogenic factors, such as VEGF, bone morphogenetic protein 4 (BMP4), plasminogen activator inhibitor-1 (PAI-1), and stem cell factor (SCF) [61–65]. As a well-characterized direct target of HIFs, VEGF stimulated the growth and migration of endothelial cells and was required for blood vessel formation [66–69]. In line with the observation that both mRNA and protein levels of VEGF were significantly induced by hypoxia in HCC cells, high VEGF expression was evident near necrotic/hypoxic regions in primary HCC samples [70]. Elevated expression of VEGF in HCC samples was correlated with early relapse and shorter survival [71, 72]. Interestingly, HIF1β only contributed partially to the hypoxic induction of VEGF in murine hepatoma cells [73], suggesting that hypoxia may employ additionally a HIF1β-independent mechanism to promote VEGF expression. Xiao H, *et al*. recently reported that H2AX phosphorylation (γ-H2AX) was required for EGFR- and HIF1α-mediated VEGF induction under hypoxia [74]. Combined evaluation of γ-H2AX, HIF1α and EGFR showed a powerful prognostic value for HCC after liver transplantation. In addition, hypoxia has been shown to induce HIF1α-dependent expression of BMP4 and HIF2α-dependent expression of PAI-1 and SCF to enhance HCC angiogenesis. Of note, despite lack of direct experimental evidence, hypoxia/HIFs-stimulated expression of erythropoietin (EPO) and platelet-derived growth factor (PDGF) may also promote HCC angiogenesis [75–78]. Therefore, HIF-mediated proangiogenic phenotypes greatly contribute to HCC angiogenesis.

### Metabolism

HIFs actively regulate a series of glycolytic genes to promote glycolysis and to favor tumor cell adaption to hypoxic stress. Many critical enzymes involved in glycolysis have been shown to be direct HIF1α targets in HCC cells, including ALDOA, GPI, GAPDH, HK2, LDHA, PGK1, PGAM1, PFKFB4, ENO1 and PKM2 [28, 79]. Moreover, glucose transporter 1 (GLUT1), responsible for cellular glucose uptake, was directly upregulated by HIF1α [80] and highly expressed in HCC cells and patient samples when compared with primary hepatocytes. High expression of GLUT1 was also associated with enhanced proliferation, poor differentiation and advanced histological stages [81]. Hexokinase 2 (HK2) and lactate dehydrogenase A (LDHA), both of which enhance glycolytic switch from glucose to pyruvate, are also direct targets of HIFs [82–84]. Moreover, HIF1α was required for the expression of pyruvate dehydrogenase kinase 1 (PDK1) which suppresses the tricarboxylic acid cycle. Ectopic PDK1 expression not only rescued the hypoxia-induced cell apoptosis, but also reduced hypoxic ROS production and restored the ATP production in HIF1α-deficient cells [85]. NDUFA4L2 was also induced by hypoxia and HIF1α. Overexpression of NDUFA4L2 was strongly associated with tumor microsatellite formation, absence of tumor encapsulation, and poor overall survival in HCC patients. Inhibition of HIF1α/NDUFA4L2 enhanced oxygen consumption and mitochondrial activity, resulting in ROS accumulation and apoptotic cell death. Depletion of NDUFA4L2 suppressed HCC xenograft growth and metastasis [86]. Interestingly, in addition to oxygen, 2-oxoglutarate, Fe (II) and ascorbate are required for proper hydroxylation of HIFα by hydroxylase, linking HIF activation to metabolic stress responses (see review [87]).

### Migration, invasion and metastasis

In HCC cells, epithelial mesenchymal transition (EMT) can be induced under hypoxia condition through the activation of Wnt/β-catenin pathway or PI3K/AKT pathway [88–90]. SNAIL1, which harbors two HREs in its promoter, can be greatly induced by HIF1α under hypoxia [91]. Hypoxia-induced miR-210 was associated with HCC cell invasion and migration through downregulating vacuole membrane protein 1 (VMP1) [92]. Hypoxia also led to the downregulation of IFT88/TG737 and promoted cell migration and invasion, partially through IFT88-mediated effects on the expression of polycystin-1, IL-8, and TGF-β1 [93]. Additional factors such as CXCL6 (HIF1α target) and SERPINB3 (HIF2α target) can promote the migratory and metastatic potentials of HCC cells [40, 94]. Moreover, Rab11-FIP4 (HIF1α target) and SCF(HIF2α target), both of which were positively associated with worse survival of HCC patients, can promote HCC cell migration and invasion *in vitro* and metastasis *in vivo* [63, 95, 96].

### Tumor microenvironment and tumor stromal cells

Hypoxic microenvironment remodels the tumor-stromal interactions in HCC. Monocytes/macrophages were recruited to the hypoxic regions of tumor tissues and subsequently up-regulated TIE2 expression [97]. These TIE2-positive monocytes/macrophages (CD14^+^CD16^+^) were proangiogenic, and their frequency in either blood or tumors correlated significantly with microvessel density in HCC [98, 99]. Moreover, hypoxia has been shown to accelerate murine HCC development by HIF1α-induced expression of IL-10 which favored the intratumoral macrophage converting from M1 to M2 type [31]. Cross-talk between hepatocyte-hepatic satellite cells also generated a proangiogenic and proinflammatory microenvironment [100]. Hypoxia-induced up-regulation of PDGF-BB in hepatic satellite cells activated the PI3K/Akt pathway in HCC cells and enhanced cell proliferation, migration, and resistance to bile acid-induced apoptosis [101].

### Cell proliferation, survival and drug/therapy response

HIF1α can directly enhance the expression of crucial oncogenes involved in cell replication in HCC, such as FoxM1 and AURKA [43, 102]. Moreover, hypoxia confers resistance to various anticancer drugs in HCC cells, including etoposide, sorafenib, SN38, cisplatin and doxorubicin [52, 78, 103–107]. Sorafenib resistant HCC patients showed higher intratumoral hypoxia [108]. Hypoxia-activated YAP and TGF-α/EGFR pathways blunted the response of HCC cells to sorafenib. Sorafenib inhibited HIF1α synthesis whereas up-regulated the expression of HIF2α, shifting hypoxic responses from HIF1α- to HIF2α-dependent pathways. Silencing of HIF2α synergized with the sorafenib to block the proliferation of HCC cells under hypoxia and the growth of xenograft tumors [109]. In addition, hypoxia-induced NDRG-1 and CBR1 can render HCC cells resistance to doxorubicin [106, 107]. Silencing of either HIF1α or HIF2α has been shown to improve the efficacy of doxorubicin in HCC models by inhibiting cell proliferation, tumor angiogenesis and enhancing cell apoptosis [53, 110]. Besides, HIF1β-deficient murine hepatoma cells were more sensitive to radiotherapy [111], suggesting that hypoxia activation may protect tumor cells from radiation. Furthermore, intratumoral gene transfer of antisense HIF1α triggered a NK cell-dependent rejection of small (0.1 cm in diameter) EL-4 tumors in mice. Combination of antisense HIF1α and B7-1-mediated immunotherapy led to a strong synergistic effect in inducing NK cell- and CD8 T cell-dependent rejection of larger EL-4 tumors (0.4 cm in diameter) [112], highlighting the promise of targeting hypoxia pathway together with cancer immunotherapy in HCC treatment.

### Therapeutic targeting of hypoxia pathway in HCC

Tremendous efforts have been made to develop effective inhibitors for HCC treatment. However, to date, sorafenib is the only FDA-approved first-line drug for advanced HCC treatment [114]. Targeted therapy for HCC is still very limited.

Given the functional importance of hypoxia pathway in HCC, targeting hypoxia related molecules may be beneficial (Table 3). Recently, several inhibitors targeting hypoxia downstream signaling (e.g. VEGF-VEGFR system) have entered phase 3 clinical trials for HCC treatment. Alternatively, the strategy of targeting HIF expression or activation is actively tested in either preclinical studies or in trials. RO7070179 and EZN-2968 are oligonucleotide inhibitors which inhibit the synthesis of HIF1α [115]. Since HIF1α translation is dependent on PI3K-AKT-mTOR pathway [116], targeted inhibition of PI3K-AKT-mTOR activity (e.g. Bufalin) effectively suppressed HIF1α expression in HCC cells [117]. ENMD-1198 (a microtubule destabilizing agent) and Metformin (an established antidiabetic drug) have also been shown to downregulate the expression of HIF1α in HCC models [118, 119]. Besides, Acriflavine which inhibits HIF1 dimerization showed anti-tumor activity in HCC models [120, 121]. Two novel small-molecule inhibitors targeting HIF2α (PT2385 and PT2977) have been evaluated in phase 1 trials in advanced clear cell renal cell carcinoma and other solid tumors [122, 123]; however, their activities against HCC cells need to be examined.

**Table 3:** Clinical trials related to targeting hypoxia pathway in HCC

Name	Mechanism of action	Disease	Clinical Stage	ClinicalTrials.gov Identifier
Apatinib	VEGFR-2 inhibitor	Hepatocellular Carcinoma	Phase 3	NCT03046979
Regorafenib	Inhibitor of multiple kinases, including VEGFR, PDGFR and FGFR	Hepatocellular Carcinoma	Phase 3	NCT01774344
Lenvatinib	Inhibitor of multiple kinases, including VEGFR	Hepatocellular Carcinoma	Phase 3	NCT01761266
Cabozantinib	VEGFR2 and MET	Hepatocellular Carcinoma	Phase 3	NCT01908426
Ramucirumab	VEGFR2	Hepatocellular Carcinoma	Phase 3	NCT02435433
RO7070179	HIF1α mRNA Antagonist	Hepatocellular Carcinoma	Phase 1	NCT02564614
EZN-2968	HIF1α antisense oligonucleotide inhibitor	Advanced Solid Tumors/ Lymphoma/Advanced Solid Tumors With Liver Metastases	Phase 1 completed	NCT02564614
OXY111A	Anti-hypoxic molecule	Hepato-Pancreato-Biliary Neoplasm	Phase 1 and 2	NCT02528526
TH-302	Hypoxia-Activated Prodrug	Advanced Kidney Cancer or Liver Cancer	Phase 1 and 2 suspend	NCT01497444
		Hepatocellular Carcinoma	Phase 1	NCT01721941
Tirapazamine	Hypoxia-Activated Prodrug	Hepatocellular Carcinoma Combined with Transarterial embolization	Phase 1	NCT02174549

Another strategy is to target hypoxia itself. OXY111A, a synthetic allosteric effector of hemoglobin to promote normoxia in hypoxic tumors, has been shown to prevent HIF1α stabilization as well as VEGF production in tumor masses [124]. OXY111A is currently under phase 1 and 2 clinical trials in patients with malignancies of the liver, pancreas and biliary tract. Moreover, hypoxia can be harnessed to selectively activate cytotoxic pro-drugs such as tirapazamine (TPZ) and TH-302 [125, 126]. By using a HBx-transgenic murine model, TPZ co-operated with arterial embolization to induce tumor necrosis without affecting normal liver cells [125]. Of note, Q6, a novel pro-drug activated under hypoxia condition, showed a more potent anti-proliferative effect than TPZ, and induced apoptosis of HCC cells. Interestingly, Q6 can also promote HIF1α degradation through autophagy pathway [127].

### Conclusions and future perspectives

As summarized above, hypoxia pathway and HIFs are involved extensively in HCC development. Although many aspects await further exploration, hypoxia pathway appears to be functionally relevant and therapeutically targetable in HCC. Further efforts can be made to characterize the mechanism of HIFs activation and putative roles of HIFs in HCC as outlined below.

As transcription factors, transactivation of HIFs in response to oxygen tension modulates a vast array of hypoxia responsive genes. However, to date, hypoxia-responsive transcriptome and the contribution of individual HIF to hypoxia response remain largely unknown in HCC. Moreover, genome-wide comparative study of various HIFs, the connections between HIFs binding and gene expression, and the contribution of epigenetic alternations in hypoxia response in HCC need to be addressed further.

Additional functional studies, including genetic models, are essential to dissect further the roles of HIFs in HCC development. Upon HIF1α inactivation, alternative pathways such as HIF2α may compensate for the HIF1α loss. HIF2α-dependent network seems to be associated more with therapy-resistance and tumor aggressiveness [108]. When compared to HIF1α, HIF2α and HIF3α remain less-well characterized in HCC. Whether HCC cells activate preferentially certain HIFs during progression is not clear, thus a more thorough investigation of the unique roles of each HIF in HCC is warranted. For example, liver-specific inactivation of HIFs using transgenic animal models with various oncogenic backgrounds may provide more insights into the function of HIFs and their interplays with different oncogenic pathways. In addition, as exemplified from HBx studies, potential impacts of hepatitis virus infection and other carcinogens on HIFs may be a fertile ground of study.

Targeting hypoxia holds a promise for HCC treatment. However, more inhibitors of HIFs and/or their co-factors need to be developed. The potential combination of hypoxia/HIF inhibitors and immunotherapy will be an exciting and active area of investigation. Further, identification of biomarkers associated with hypoxia-targeted therapy will be very valuable and helpful.
